# Atypical antipsychotics as add-ons to antidepressants in the major depressive disorder – A risks and benefits analysis

**DOI:** 10.1192/j.eurpsy.2025.2105

**Published:** 2025-08-26

**Authors:** O. Vasiliu

**Affiliations:** 1 Neuroscience Department, “Carol Davila” University of Medicine and Pharmacy; 2 Psychiatry Department, “Dr. Carol Davila” University Emergency Central Military Hospital, Bucharest, Romania

## Abstract

**Introduction:**

Major depressive disorder (MDD) is associated with high rates of incomplete response during treatment, with only one-third of the patients reaching remission after the first trial of antidepressants, according to the STAR*D trial. Based on the results of the same study, only 67% of the patients with MDD obtained remission after four trials of antidepressants, including a monoamine oxidase inhibitor, i.e., tranylcypromine, and various augmenting strategies. However, in STAR*D, no atypical antipsychotics (AAPs) were used, which is an important shortcoming of this trial. Exploring the efficacy of AAPs as add-on agents to antidepressants in the case of MDD with partial responsiveness may improve the prognosis of patients who did not remit during either antidepressant monotherapy or antidepressant therapy augmented with other psychotropic agents.

**Objectives:**

To assess the available data on the efficacy and tolerability of atypical antipsychotic augmentation in patients with MDD who obtained only partial response to antidepressants.

**Methods:**

This review included three databases (Google Scholar, PubMed, and EMBASE) that were searched from their inception until June 2024 for papers published in English corresponding to the keywords “major depressive disorder,” and “partial response” and “atypical antipsychotics”. Both primary and secondary reports were included.

**Results:**

Systematic reviews dedicated to this topic reported significantly superior responses to placebo for ziprasidone, risperidone, aripiprazole, brexpiprazole, cariprazine, and quetiapine when added to antidepressants. The tolerability of these augmenting agents was low, with high rates of early treatment discontinuation reported. Only risperidone was reported in a systematic review as similar to placebo in terms of tolerability for this population. Network meta-analyses showed positive results for quetiapine, olanzapine, aripiprazole and brexpiprazole in terms of efficacy, but in terms of acceptability, no difference between these four antipsychotics and between each of them and placebo were found; tolerability was low for all antipsychotics vs. placebo, but the certainty of most evidence was evaluated as low and very low. Aripiprazole, quetiapine, olanzapine, brexpiprazole and cariprazine are approved by the FDA for the adjunctive treatment of MDD that does not respond to antidepressant monotherapy.

**Conclusions:**

AAPs are an efficient option for augmenting antidepressants when MDD is only partially responsive to antidepressants, but careful monitoring of this strategy’s tolerability is needed, and high rates of treatment discontinuation are reported.

**Disclosure of Interest:**

None Declared

